# Supplementary material for: Combined Proteomics and Metabolism Analysis Unravels Prominent Roles of Antioxidant System in the Prevention of Alfalfa (Medicago sativa L.) against Salt Stress
Source: Int J Mol Sci. 2020 Jan 30;21(3):909. doi: 10.3390/ijms21030909 (PMC7037825; doi:10.3390/ijms21030909)
Supplement: Supplementary file 1 [file ijms-21-00909-s001.zip › Suppl materials/Supplementary Figures.docx]

**Supplementary Figures**


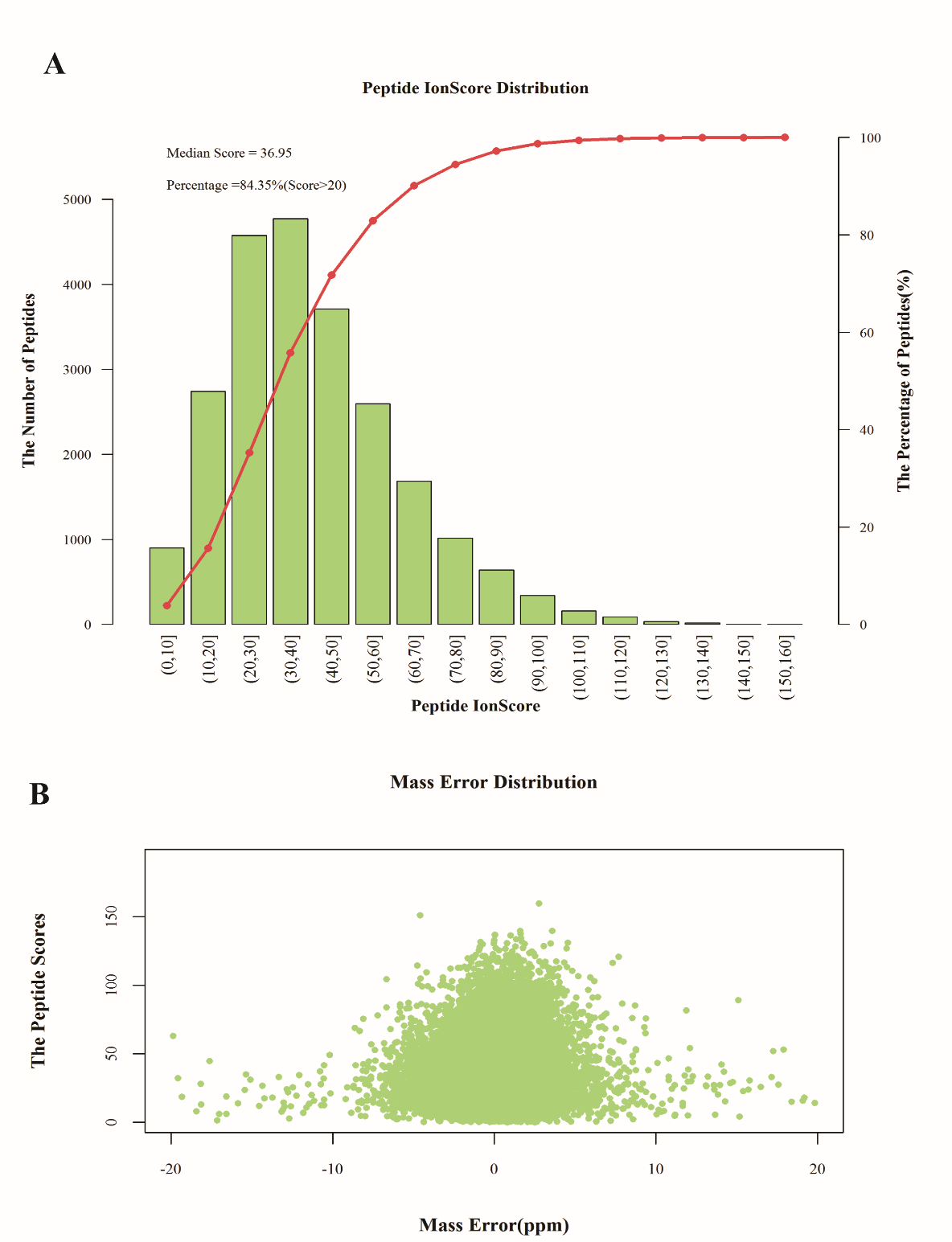


**Figure S1.** The numbers of proteins versus peptide IonScore and mass error distributions. A, Histogram plot representing the numbers and percentage of peptides against IonScore. The x-axis represents the number of peptides identifying the protein; the left y-axis represents the number of proteins corresponds to the histogram bars. The right y-axis indicates the cumulative curve in the graph. B, Scatter plots representing the peptide score against mass error.


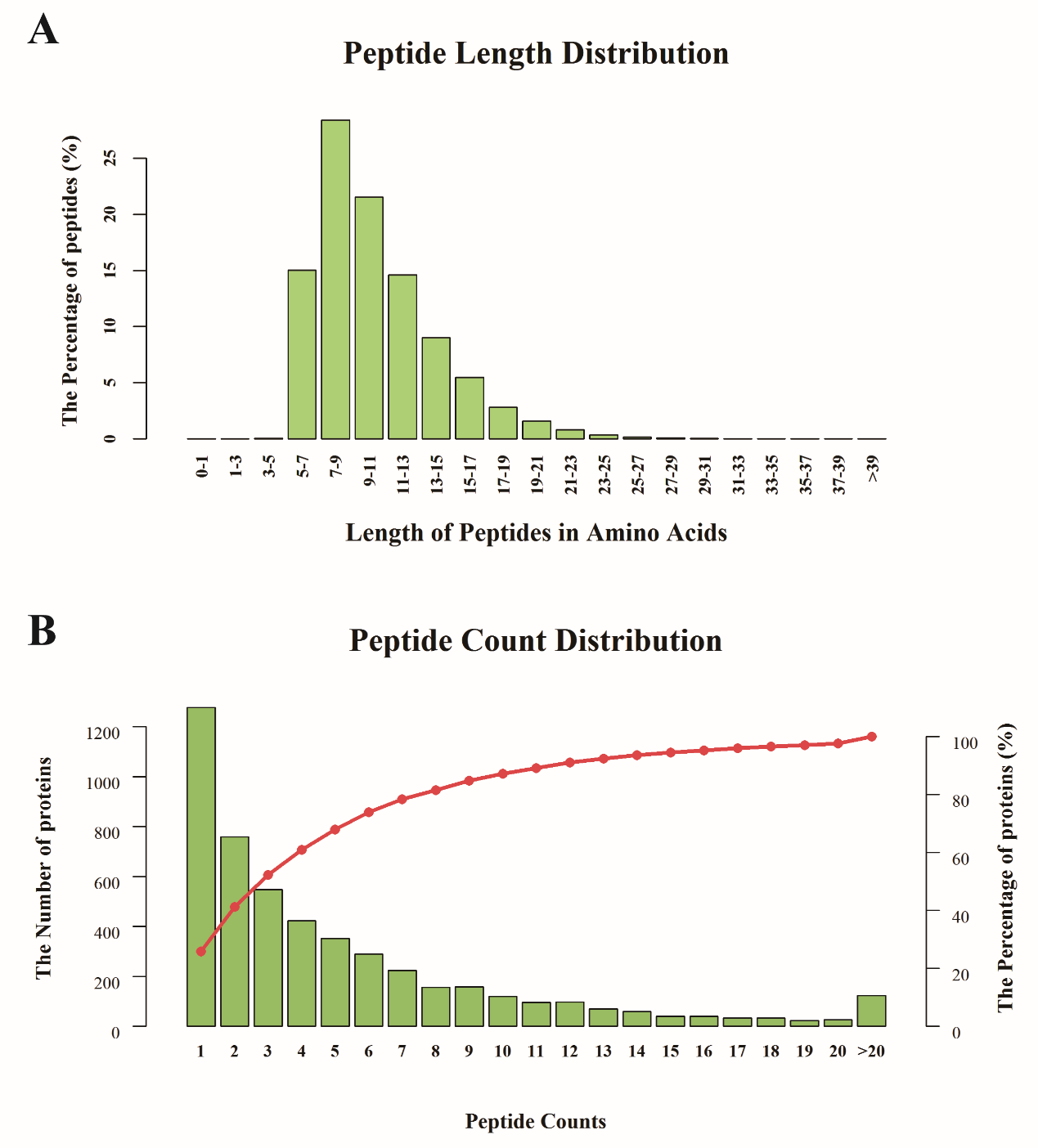


**Figure S2.** The numbers of peptides versus their length distribution and count distribution. A, Histogram plot representing the distribution of peptide length among different length of peptides in amino acids. The x-axis represents the number of amino acids in the identified peptide sequence. The y-axis represents the number of peptides (in percentage) identified. B, Histogram plot representing the numbers of proteins among different peptide count. The figure shows the distribution of the number of identified peptides corresponding to the identified protein.

**
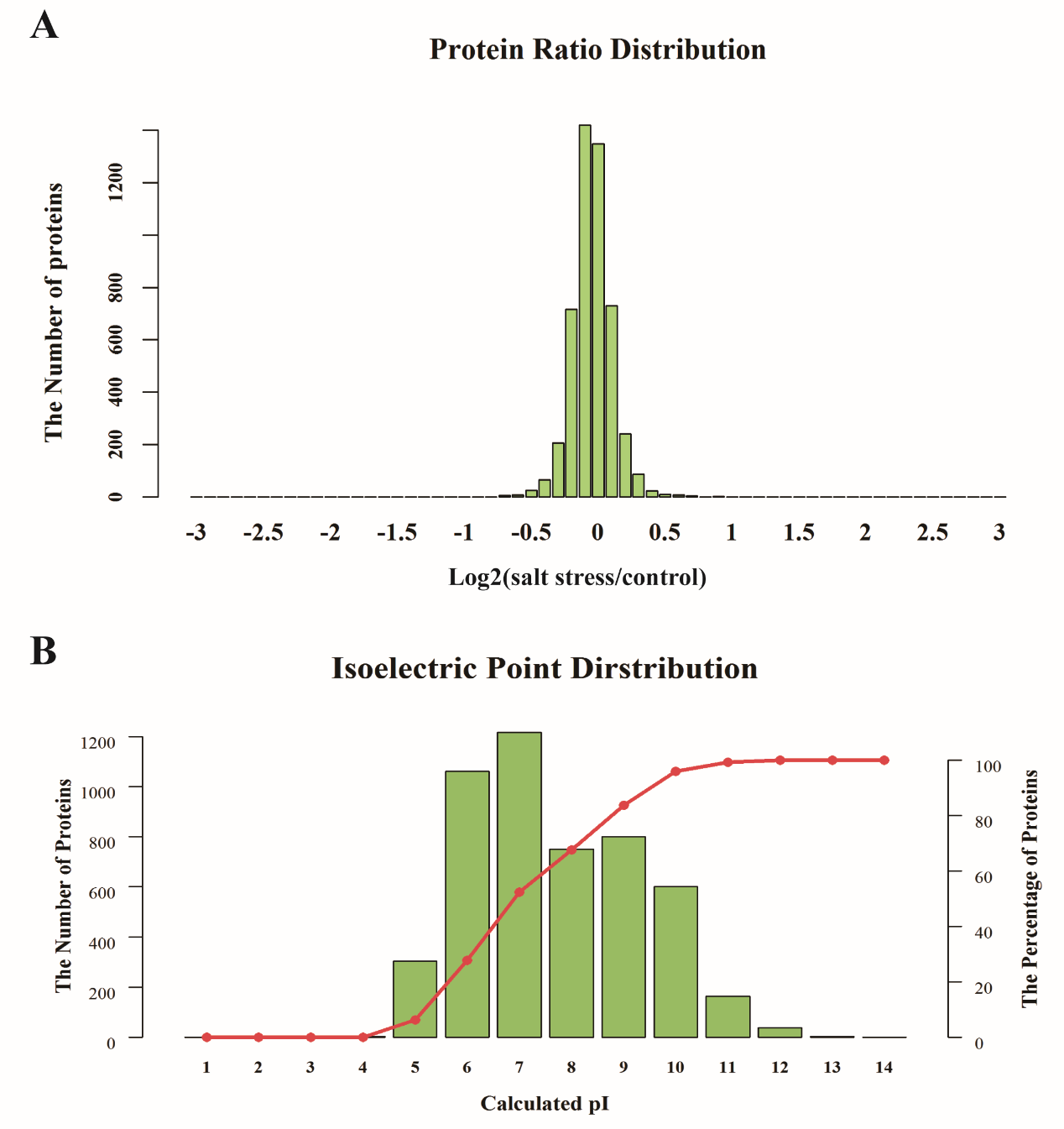
**

**Figure S3.** The numbers of proteins versus their ratio distribution and isoelectric point distribution. A, Histogram representing the numbers of proteins against the differential abundant proteins under 200 mM NaCl treatment by control (200 mM/control). The x-axis represents the difference multiplier (base 2 logarithmic transformations) and y-axis indicates the number of proteins identified. B, The numbers of proteins against calculated isoelectric point (pI). The left y-axis represents the number of proteins and the right y-axis depicts the cumulative curve in the percentage of proteins. The electrical point is not higher than the cumulative percentage of protein of the corresponding value.
